# Statistical models versus machine learning approach for competing risks in proctological surgery

**DOI:** 10.1007/s13304-025-02109-0

**Published:** 2025-01-25

**Authors:** Lucia Romano, Andrea Manno, Fabrizio Rossi, Francesco Masedu, Margherita Attanasio, Fabio Vistoli, Antonio Giuliani

**Affiliations:** 1 Department of Biotechnological and Applied Clinical Sciences, University of L’Aquila, L’Aquila, Italy; 2 Department of Information Engineering, Computer Science and Mathematics, University of L’Aquila, L’Aquila, Italy; 3 Center of Excellence DEWS, University of L’Aquila, L’Aquila, Italy

**Keywords:** Competing risks, Predictive performance, Logistic regression, Supervised machine learning

## Abstract

Clinical risk prediction models are ubiquitous in many surgical domains. The traditional approach to develop these models involves the use of regression analysis. Machine learning algorithms are gaining in popularity as an alternative approach for prediction and classification problems. They can detect non-linear relationships between independent and dependent variables and incorporate many of them. In our work, we aimed to investigate the potential role of machine learning versus classical logistic regression for the preoperative risk assessment in proctological surgery. We used clinical data from a nationwide audit: the database consisted of 1510 patients affected by Goligher’s grade III hemorrhoidal disease who underwent elective surgery. We collected anthropometric, clinical, and surgical data and we considered ten predictors to evaluate model-predictive performance. The clinical outcome was the complication rate evaluated at 30-day follow-up. Logistic regression and three machine learning techniques (Decision Tree, Support Vector Machine, Extreme Gradient Boosting) were compared in terms of area under the curve, balanced accuracy, sensitivity, and specificity. In our setting, machine learning and logistic regression models reached an equivalent predictive performance. Regarding the relative importance of the input features, all models agreed in identifying the most important factor. Combining and comparing statistical analysis and machine learning approaches in clinical field should be a common ambition, focused on improving and expanding interdisciplinary cooperation.

## Introduction

Clinical risk prediction models are ubiquitous in many surgical domains. The traditional approach to develop these models involves the use of regression analysis, for example, logistic regression (LR) to predict disease outcomes (prognosis) after surgical interventions. Machine learning (ML) algorithms are gaining in popularity as an alternative approach for prediction and classification problems. In fact, advances in processing power and cloud storage have given clinicians access to increased amounts and types of data, that facilitated the utilization of artificial intelligence (AI). ML is a component of AI; it relies on computer algorithms and data analysis to learn patterns that exceeds the capacity of the human mind to comprehend [1]. They can detect non-linear relationships between independent and dependent variables and incorporate many of them [2, 3]. In clinical settings, for risk stratification of patients, various types of supervised machine learning algorithms have been used with large clinical databases. “Supervised” refers to the existence of a training set: in these algorithms, part of the data set (the “training” data set) is analyzed to build a model, and another part of the data set (the “testing” data set) is used to validate the model [4].

Although both techniques (LR and ML) have been used to develop risk models for postoperative complications, it is unclear if machine learning is superior to logistic regression when using structured data. Our study used clinical data from a nationwide audit [5] to compare machine learning to logistic regression in predicting complications after planned surgery for hemorrhoidal disease. We investigated the application of three ML supervised algorithms (Decision Tree, Support Vector Machine, XGBoost) to provide a comprehensive evaluation for the problem. The predictive value of the considered models was compared using AUC (area under the curve), balanced accuracy, sensitivity, and specificity.

## Methods

### Source of data

We performed a multicenter retrospective observational study including patients affected by Goligher’s grade III hemorrhoidal disease (HD) who underwent hemorrhoidal artery ligation (HAL) with mucopexy or excisional hemorrhoidectomy (EH) between January 2016 and February 2020. Any center belonging to the Italian Society of Colorectal Surgery (SICCR) in which at least 30 surgical procedures per year for hemorrhoidal disease were performed was able to join the study.

The study was conducted in accordance with the Declaration of Helsinki (1996) and International Conference on Harmonization-Good Clinical Practice (ICHGCP) guidelines, and it obtained approval from local ethics committee (Prot. n. 51,380, 22.04.2021). It was registered on ClinicalTrials.gov (Identifier: NCT04863963). Patients selected for the study gave informed consent to participate.

### Participants

We included patients aged 18 years or older, with Goligher’s grade III HD, who underwent elective conventional excisional hemorrhoidectomy (EH) or transanal dearterialization (HAL) with or without use of the Doppler transducer and with mucopexy, for whom a 30-day follow-up was available. Exclusion criteria were as follows: recurrent disease; presence of Crohn’s disease or ulcerative colitis; coagulopathies. We also excluded cases in which combined surgical procedures were performed and cases in which the procedure did not correspond to those described below. Diagnosis of primary HD was established by clinical examination and anoscopy or proctoscopy.

### Data collection

Information was stored in an online database. Collected data included patients’ demographics and clinical information, operative details such as procedure and anesthesia; postoperative complications. Patients were asked to report their symptoms in the preoperative period, using a validated questionnaire [6] specifically designed to assess the severity of hemorrhoidal symptoms using five different parameters (bleeding, prolapse, manual reduction, discomfort/pain, impact on QoL), and based on a five points scale from 0 (never) to 4 (with every bowel movement). Complications were evaluated according to the Clavien–Dindo classification [7].

## Statistical analysis

The dataset contained ten prognostic factors. Eight were qualitative: sex, presence of rectal mucosal prolapse, preoperative prolapse, preoperative bleeding, preoperative manual reduction, preoperative pain/discomfort, surgical treatment performed, type of anesthesia (general, spinal or local). Two were quantitative: age and preoperative score (from 0 to 24).

Qualitative data were expressed as numerical values and percentages, and comparison between groups was performed using the χ^2^ test or Fisher’s exact test, as appropriate. Quantitative variables were described as mean and standard deviation (SD) and median and range, and the comparison between groups was conducted using two-sample Wilcoxon rank-sum (Mann–Whitney) test. Statistical significance was set at *p* < 0.05.

Dataset was first randomly split into a “train” group (80% of the whole dataset), for the development and validation of models, and a “test” group (20% of the whole dataset) used for making predictions. This data split was fixed and used for all models.

Since the dataset was highly imbalanced (the number of samples without complications is much larger than the number of samples with complications), we have applied the well-known SMOTE method [8] to address the class imbalance. To set the hyperparameters of each ML model (i.e., the parameters which cannot be tuned during the training phase), we have applied a grid-search cross-validation technique [9].

For evaluating model performance, we considered:

- True positives (TP)
- True negatives (TN)
- Test accuracy = (TP + TN)/all patients
- Sensitivity (true positive rate, TPR) = TP/(TP + FN)
- Specificity (true negative rate, TNR) = TN/(TN + FP)
- Balanced accuracy = average between the sensitivity and the specificity
- Precision (positive predictive value, PPV) = TP/(TP + FP)
- Negative predictive value (NPV) = TN/(TN + FN)
- The area under the receiver operating characteristic (ROC) curve (AUC).

A graphical visualization of the performance of the models was represented by confusion matrices.

Moreover, we applied the widely used SHAPLEY values analysis [10] to enhance the explainability of the predictions of ML models.

### Logistic regression model (LR)

The associations between the predictors and outcomes were displayed as odds ratios (ORs), 95% confidence intervals (CIs), and *p* values. Backward stepwise selection with the Akaike and Bayesian information criterion (AIC and BIC) was used to choose the best model. The optimal cut-off value of *p* (the probability for a patient that a post-operative complication occurs) was calculated using a ROC curve analysis and the Youden index. Analyses were conducted using R software (version 4.1.2).

### Machine learning techniques (ML)

Decision Tree (DT), Support Vector Machine (SVM), and Extreme Gradient Boosting (XGB) algorithms [11–14] were used to assess the predictive weight of prognostic factors on the occurrence of complications. DT and SVM were implemented using the Python sklearn package, while XGB was taken from https://xgboost.readthedocs.io/en/stable. All the experiments have been carried out on a laptop Intel(R) Core(TM) i7-6700HQ CPU@2.60G with 8 GB of RAM.

## Results

Twenty-eight centers belonging to the Italian Society of Colorectal Surgery (SICCR) joined the study. The database consisted of 1731 total patients who underwent elective surgery between January 2016 and February 2020, of which 1681 met the defined criteria. The dataset contained 171 (10%) missing data overall for the variables, with 1510 complete cases (90%) that were included in the analysis. The mean age of all patients (59.7% males and 40.3% females) was 53 years (SD: 13.0).

Based on the Clavien–Dindo classification, we considered grades 0 and I as “no complications” and grades II, III, IV, and V as “presence of complications”. According to this definition, ten per cent (10%) of patients reported complications (148 patients out of 1510). Characteristics of the sample stratified by complications are reported in Table 1.

**Table 1:** Demographic and clinical characteristics of the sample stratified by complications yes/no

Variables	No complications (*n* = 1362)	Complications (*n* = 148)	*p* value
Sex, *n* (%)			< 0.001**
Male	838 (61.5)	63 (42.6)	
Female	524 (38.5)	85 (57.4)	
Age, years			0.006***
Mean (SD)	53 (13.1)	50 (12.2)	
Median (range)	52 (20–90)	49 (24–81)	
Mucosal prolapse, *n* (%)			< 0.001**
Yes	1056 (77.5)	139 (93.9)	
No	306 (22.5)	9 (6.1)	
Pre-op. prolapse, *n* (%)			0.024**
Yes	1119 (82.2)	139 (93.9)	
No	243 (17.8)	9 (6.1)	
Pre-op. bleeding, *n* (%)			< 0.001**
Yes	1212 (89.0)	146 (98.6)	
No	150 (11.0)	2 (1.4)	
Pre-op. manual reduction, *n* (%)			< 0.001**
Yes	841 (61.7)	136 (91.9)	
No	521 (38.3)	12 (8.1)	
Pre-op. pain/discomfort, *n* (%)			0.004**
Yes	1255 (92.1)	146 (98.6)	
No	107 (7.9)	2 (1.4)	
Pre-op. score			< 0.001***
Mean (SD)	10.4 (4.4)	14.5 (3.8)	
Median (range)	11 (0–24)	15 (2–20)	
Treatment, *n* (%)			< 0.001**
EH*	996 (73.1)	132 (89.2)	
HAL*	366 (26.9)	16 (10.8)	
Anesthesia, *n* (%)			< 0.001**
General	116 (8.5)	4 (2.7)	
Spinal	1048 (76.9)	29 (19.6)	
Local	198 (14.5)	115 (77.7)	

*EH excisional hemorrhoidectomy, *HAL* hemorrhoidal artery ligation

**χ2 test or Fisher’s exact test

***Two-sample Wilcoxon rank-sum (Mann–Whitney) test

We can see from Table 1 that all the collected variables were statistically significant among groups “complications” and “no-complications” at the univariate analysis, so they could be all included in the regression analyses. However, based on the structure of the administered questionnaire, we reported the occurrence of collinearity among the variable “preoperative score” and the variables “preoperative prolapse”, “preoperative bleeding”, “preoperative manual reduction”, and “preoperative pain/discomfort”. Backward stepwise selection showed that the choice of variable “preoperative score” reduced both parameters AIC and BIC, so we decided for a model that included this variable and excluded the other four.

Then the dataset was randomly split into “train” and “test” groups, and the characteristics of the sample stratified by group are reported in Table 2.

**Table 2:** Demographic and clinical characteristics of the sample stratified by group (train or test)

Variables	Train (*n* = 1208)	Test (*n* = 302)	*p* value
Sex, *n* (%)			0.416**
Male	727 (60.2)	174 (57.6)	
Female	481 (39.8)	128 (42.4)	
Age, years			0.304***
Mean (SD)	53 (13.1)	52 (12.7)	
Median (range)	52 (20–90)	52 (27–88)	
Mucosal prolapse, n (%)			0.874**
Yes	955 (79.1)	240 (79.5)	
No	253 (20.9)	62 (20.5)	
Pre-op. score			0.526***
Mean (SD)	10.8 (4.4)	11.0 (4.7)	
Median (range)	11.0 (1–24)	11.0 (0–20)	
Treatment, n (%)			0.723**
EH*	900 (74.5)	228 (75.5)	
HAL*	308 (25.5)	74 (24.5)	
Anesthesia, n (%)			0.656**
General	90 (7.5)	30 (9.9)	
Spinal	870 (72.0)	207 (68.5)	
Local	248 (20.5)	65 (21.5)	
Complications, n (%)			0.931**
Yes	118 (9.8)	30 (9.9)	
No	1090 (90.2)	272 (90.1)	

*EH excisional hemorrhoidectomy, *HAL* hemorrhoidal artery ligation

**χ^2^ test or Fisher’s exact test

**Two-sample Wilcoxon rank-sum (Mann–Whitney) test

In this case, differences among variables were not statistically significant between the two groups, which resulted, therefore, to be comparable.

Logistic regression analysis performed on the “train” group is showed in Table 3.

**Table 3:** Multivariate logistic regression analysis of predictors for complications

Predictor	Estimate	Odds ratio	95% confidence interval		*p* value
			Lower	Upper	
Intercept	− 6.86	0.001	2.42e-4	0.005	< .001
Age	− 0.03	0.975	0.9	0.993	0.006
Sex	0.33	1.397	0.892	2.187	0.144
Mucosal prolapse	1.33	3.768	1.609	8.820	0.002
Treatment	− 0.55	0.579	0.311	1.076	0.084
Anesthesia	2.31	10.103	6.326	16.133	< .001
Spinal anesthesia	− 0.30	0.738	− 1.555	0.948	0.635
Local anesthesia	2.27	9.710	1.039	3.507	< .001
Pre-operative score	0.12	1.128	1.056	1.204	< .001

**Fig. 1:**
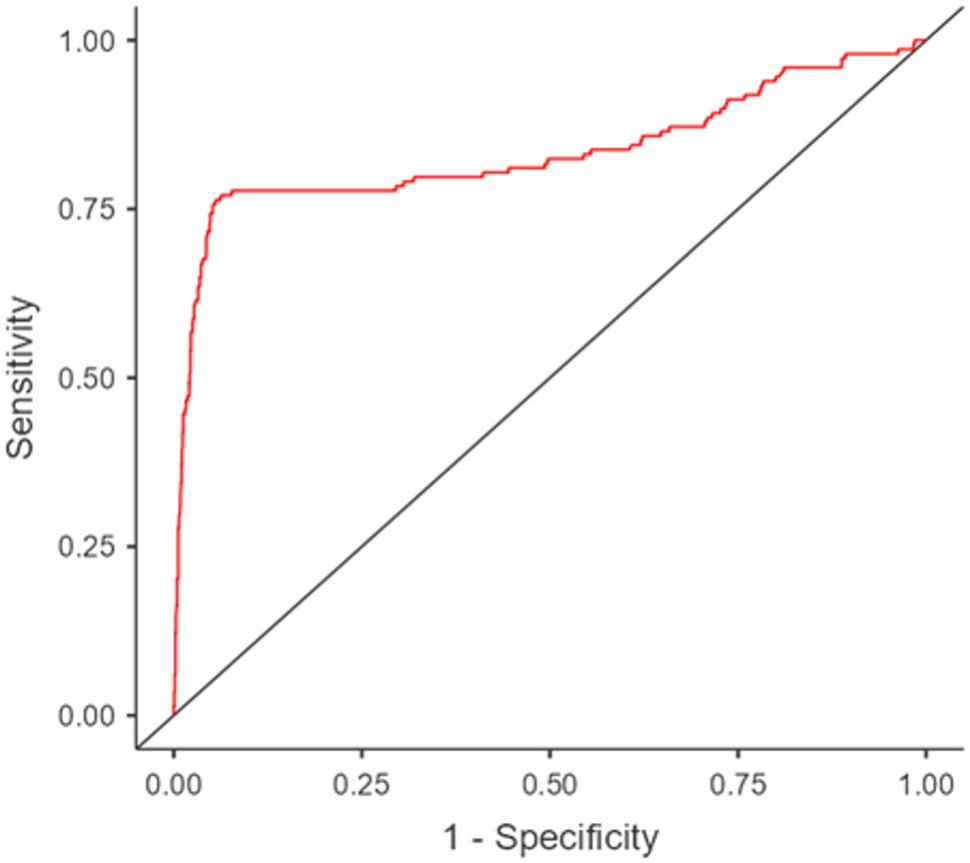
ROC curve of logistic regression model

The AUC was 0.83 (Fig. 1) and the optimal cut-off value of p defined by the Youden index was 0.24. This model was then performed on the “test” database (302 patients, of which 30 reported complications), with the performance metrics reported in Table 4.

**Table 4:** Comparison among model performances

	Logistic regression model *	Decision Tree	Support Vector Machine	XGBOOST
P	30	30	30	30
N	272	272	272	272
TP	25	24	26	25
TN	250	241	222	254
Test Accuracy	91%	88%	82%	92%
Balanced test accuracy	88%	84%	84%	88%
ROC AUC score	0.83	0.84	0.84	0.88
TPR (sensitivity)	83%	80%	87%	80%
TNR (specificity)	92%	89%	82%	93%
PPV (precision)	53%	44%	34%	58%
NPV	98%	98%	98%	98%

*P* positive, *N* negative, *TP* true positives, *TN* true negatives, *TPR* true positives rate, *TNR* true negatives rate, *PPV* positive predictive value, *NPV* negative predictive value

*Cut-off value of *p* = 0.24

**Fig. 2:**
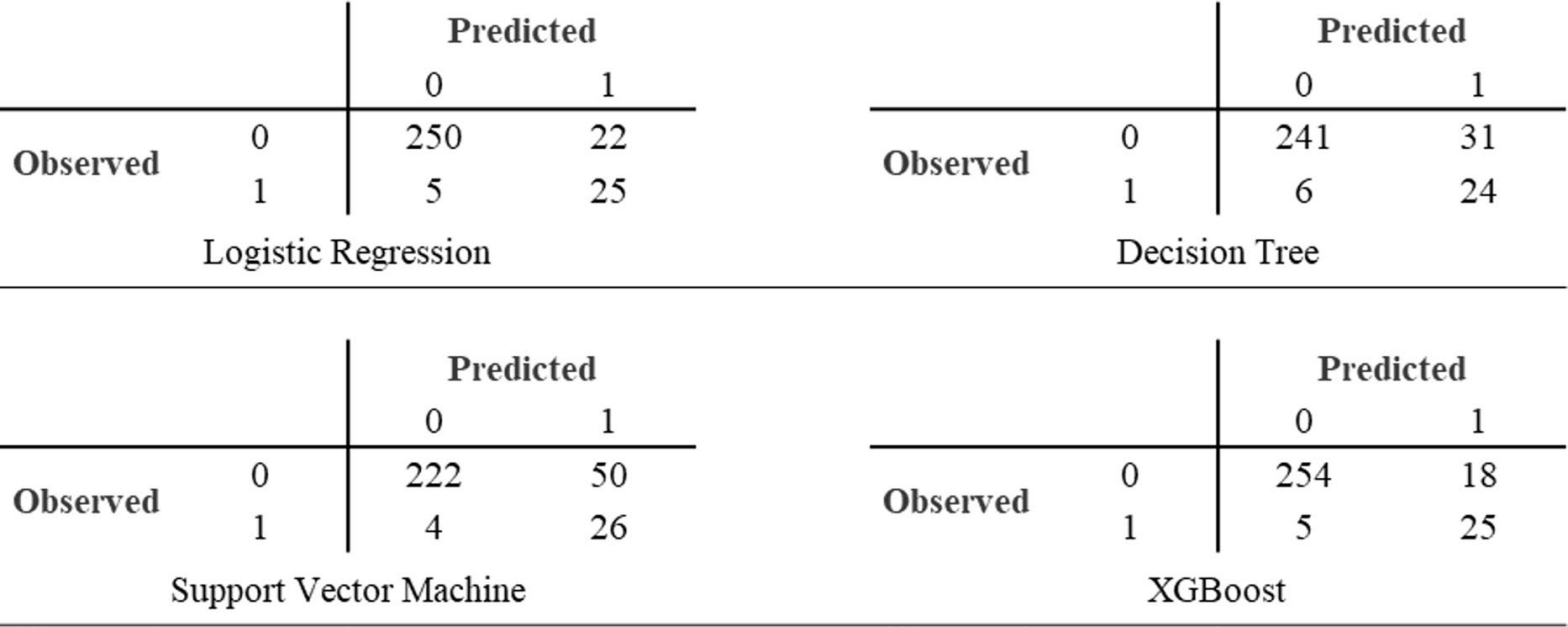
Confusion matrices of the included models

Decision Tree (DT), Support Vector Machine (SVM), and XGBoost (XGB) algorithms were then performed. Confusion matrices of each model is reported in Fig. 2, and discrimination performance of different models was finally compared (Table 4).

The AUC of the multivariate logistic regression model was 0.83, and the balanced test accuracy was 88%, with sensitivity and specificity of 0.83 and 0.92, respectively.

The AUC of the DT model was 0.84, and the balanced test accuracy was 84%, with sensitivity and specificity of 0.80 and 0.89, respectively.

The AUC of the SVM model was 0.84, and the balanced test accuracy was 84%, with sensitivity and specificity of 0.87 and 0.82, respectively.

The AUC of the XGBoost model was 0.88, and the balanced test accuracy was 88%, with sensitivity and specificity of 0.80 and 0.93, respectively.

The models also reported the input features importance to establish the importance of each feature in the competing risk assessment, except for the SVM algorithm which could not provide this information. For LR analysis, feature importance was reported in terms of odds ratio (OR) and *p* value for significance; for ML models, it was reported in terms of relative importance (the higher the score for a feature, the larger effect it has on the model to predict a certain variable).

According to LR model, the three most important and significant variables that led to increased surgical risk were:

- Anesthesia (OR 10.1, *p* < 0.001)
- Mucosal prolapse (OR 3.8, *p* = 0.002)
- Preoperative score (OR 1.1, *p* < 0.001)

According to DT, the three most important variables that led to increased surgical risk were:

- Anesthesia
- Pre-operative score
- Treatment

According to XGB, the three most important variables that led to increased surgical risk were:

- Pre-operative score
- Anesthesia
- Treatment

**Fig. 3:**
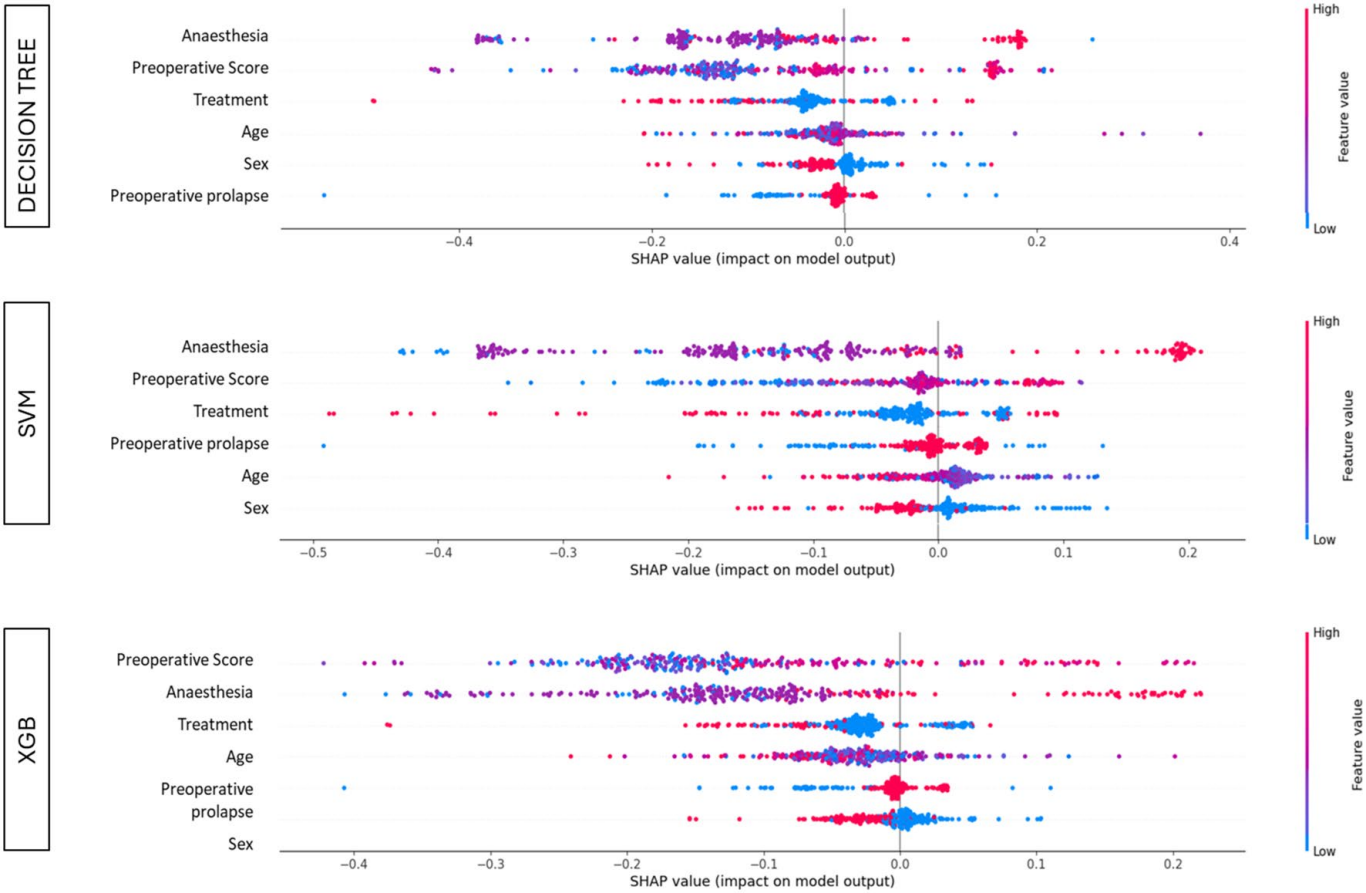
SHAPLEY values analysis of ML models

SHAPLEY values of ML models are reported in Fig. 3. Notice that this allows to have a feature ranking also for the SVM model, for which no feature importance is structurally provided. In such plots, for each feature, all samples of the dataset are horizontally distributed according to their SHAPLEY values, so that the larger is the range of the horizontal distribution, the stronger is the impact of that feature on the final prediction (occurrence of complications). Features are ranked from the top (most important) to the bottom (less important). Moreover, the color of each sample marker allows to detect if a feature value tends to influence positively (i.e., toward “complication”) or negatively (toward “no complications”) the associated prediction.

## Discussion

To the best of our knowledge, this is the first research comparing classical statistical methods (LR) with ML techniques for predicting complications after surgery for hemorrhoidal disease. Among ML techniques, we decided to use DT and SVM because they are the most employed models for clinical risk prediction [15], and we also performed XGB because it is an ensemble method with improved predictive performance than single ones. We have used the same dataset to train all our models: this is crucial for valid model comparisons.

Our study found no superiority of the ML using structured pre- and intra-operative variables. In detail, the results showed that among the ML techniques, XGB was the most complex and accurate; however, it overlapped with LR in terms of balanced accuracy, specificity, and negative predictive value (NPV). The AUC and precision were slightly better in XGB than LR, but on the other hand, LR had a higher sensitivity, which means that it has the ability to better predict high risk patients. In the other ML models (DT and SVM), the performance metrics were on average weaker with respect to both LR and XGB.

These results are consistent with several recent studies and reviews [3, 15–23] on the comparative performance of machine learning and logistic regression in different clinical applications. For example, Zhen et al. [18] observed in their dataset containing 733 women diagnosed with preeclampsia that machine learning algorithms can accomplish prediction modeling and demonstrate superior discrimination, while logistic regression can be well calibrated. Lee et al. [19] applied different ML models on a dataset of 1211 patients and found that gradient boosting machine showed the best performance for the prediction of acute kidney injury after liver transplantation. Lynam et al. [21] concluded that logistic regression performed as well as optimized machine algorithms to classify patients with diabetes, using a dataset of 1378 participants and 7 predictor variables. Finally, a recent review conducted by Christodoulou et al. [15] including 71 studies with a median sample size of 1250 patients found no evidence of superior performance of ML over LR. Other recent studies reported in detail that the AUCs of machine learning techniques were not superior to logistic regression models to predict postoperative mortality [22, 23].

Regarding the relative importance of the input features, all models agreed in identifying anesthesia and pre-operative score as the most important factors. In particular, from LR in terms of OR emerged that local anesthesia was about ten times more at risk than general anesthesia (*p* < 0.001), while spinal anesthesia was associated with a lower risk, although this data was not statistically significant (*p* = 0.635). We can see that LR associates a coefficient to every feature representing its influence on the final prediction, and this makes it easily interpretable. In contrast, for ML models, there is the well-known problem of poor interpretability (black-box phenomenon: high accuracy, but low transparency and interpretability for humans), although these algorithms have actually stronger representational power and predictive capabilities [2, 3].

To bridge this gap, we tried to use the SHAPLEY values analysis which gives us a graphic representation of the model predictions and helps us to understand the directions of predictions (toward increasing or reducing risk).

Our study has several limitations. The first one is that the number of patients in this database may have been too small for machine learning to show its benefit. In fact, to successfully train and use ML models, the dataset on which they are trained needs to be sufficiently large [24]. However, in this first phase of the study, we made a methodological choice to control confounding factors. We tried to select a controlled and homogeneous sample, using a clinical sample in which the data recruiting has been taken care of as quality: they are not self-administered questionnaires, but there is clinical information reported by researchers. In this way, there is control of the input information, to obtain more controlled results. The aim of our study was to explore and compare the performance of machine learning and a classic statistical modeling approach; in this view, the sample used should be unbiased. Bias control is achieved with the study design and the inclusion criteria. More restrictive criteria obviously reduce generalizability, but they guarantee a higher level of internal validity. Moreover, clinical settings are often characterized by low-medium sample size and limited number of predictors, as confirmed by recent clinical studies in the literature, which apply ML techniques to similar or even much smaller datasets [25–28].

The use of only seven predictors may be considered the second limitation of our study, since these machine learning algorithms are designed to deal with larger datasets and more variables. However, working with few meaningful predictors is common in clinical practice, and knowing the performance of machine learning models in these settings is important. The use of only seven predictor variables also means a very low risk of over-fitting (too many predictors and too much complexity relative to few outcome events) [29].

With regard to variables, it is also important to consider the limit related to the omitted variable bias: the results tend to characterize the association profile between complications and selected covariates. There are many potential confounders, among which is the patients’ comorbidities. However, in our analysis, the type of variable pool emphasized the predictive role of pre- and intra-operative factors, which are potentially modifiable predictors. Taking into account modifiable factors, risk prediction may be followed by improved patient outcomes.

## Conclusion

Statistical analysis and machine learning are two similar scientific domains, and their predictive abilities can vary according to the characteristics of datasets. We used a medium sample size, a limited number of predictors, and mostly discrete variables. In this setting, advanced ML and classical LR models reached an equivalent predictive performance. However, a larger number of medical records is still needed for further comparison between models, and for evaluation of the algorithmic applicability to structural medical records.

We are aware of the role of ML as a powerful analytical tool that can be even superior to traditional methods depending on the complexity and nature of the data. For this purpose, combining and comparing statistical analysis and machine learning approaches in clinical field should be a common ambition, focused on improving and expanding interdisciplinary cooperation. In fact, ML can offer a powerful and comprehensive approach in the right contexts, but further validation procedures are needed to define situations where modern methods have advantages over traditional approaches.

## Declarations

### Data availability statement

The data that support the findings of this study are not openly available due to reasons of sensitivity and are available from the corresponding author upon reasonable request.
